# How experience with tone in the native language affects the L2 acquisition of pitch accents

**DOI:** 10.3389/fpsyg.2022.903879

**Published:** 2022-08-19

**Authors:** Katharina Zahner-Ritter, Tianyi Zhao, Marieke Einfeldt, Bettina Braun

**Affiliations:** ^1^Department of Phonetics, University of Trier, Trier, Germany; ^2^Department of Linguistics, University of Konstanz, Konstanz, Germany

**Keywords:** second language acquisition, proficiency, tone, intonation, alignment, experience, imitation, general additive mixed models

## Abstract

This paper tested the ability of Mandarin learners of German, whose native language has lexical tone, to imitate pitch accent contrasts in German, an intonation language. In intonation languages, pitch accents do not convey lexical information; also, pitch accents are sparser than lexical tones as they only associate with prominent words in the utterance. We compared two kinds of German pitch-accent contrasts: (1) a “non-merger” contrast, which Mandarin listeners perceive as different and (2) a “merger” contrast, which sounds more similar to Mandarin listeners. Speakers of a tone language are generally very sensitive to pitch. Hypothesis 1 (H1) therefore stated that Mandarin learners produce the two kinds of contrasts similarly to native German speakers. However, the documented sensitivity to tonal contrasts, at the expense of processing phrase-level intonational contrasts, may generally hinder target-like production of intonational pitch accents in the L2 (Hypothesis 2, H2). Finally, cross-linguistic influence (CLI) predicts a difference in the realization of these two contrasts as well as improvement with higher proficiency (Hypothesis 3, H3). We used a delayed imitation paradigm, which is well-suited for assessing L2-phonetics and -phonology because it does not necessitate access to intonational meaning. We investigated the imitation of three kinds of accents, which were associated with the sentence-final noun in short *wh*-questions (e.g., *Wer malt denn Mandalas*, lit: “Who draws PRT mandalas?” “Who likes drawing mandalas?”). In Experiment 1, 28 native speakers of Mandarin participated (14 low- and 14 high-proficient). The learners’ productions of the two kinds of contrasts were analyzed using General Additive Mixed Models to evaluate differences in pitch accent contrasts over time, in comparison to the productions of native German participants from an earlier study in our lab. Results showed a more pronounced realization of the non-merger contrast compared to German natives and a less distinct realization of the merger contrast, with beneficial effects of proficiency, lending support to H3. Experiment 2 tested low-proficient Italian learners of German (whose L1 is an intonation language) to contextualize the Mandarin data and further investigate CLI. Italian learners realized the non-merger contrast more target-like than Mandarin learners, lending additional support to CLI (H3).

## Introduction

Acquiring a second language (L2) poses many concurrent challenges for the learner: building a lexicon, getting the syntax right, and producing segmental and suprasegmental elements of the language correctly. This paper focuses on the acquisition of prosodic aspects, namely pitch accents in an intonation language. The acquisition of intonation in the L2 is influenced by a large range of factors, such as the native language/variety (for overview, see Mennen, 2015; Trouvain and Braun, 2021), proficiency (Grabe et al., 2003; So and Best, 2010; He et al., 2012; Graham and Post, 2018; Shang and Elvira-García, 2022), musical abilities (Li et al., 2022), language aptitude (Jilka, 2009), etc. Here, we study the roles of *native language* and *proficiency* in the acquisition of L2 intonation. As a test case, we examine how native speakers of a tone language (L1: Mandarin), who are low- or high-proficient learners of an intonation language (L2: German), acquire German pitch accents. Given the prosodic differences between Mandarin and German, this allows us to investigate the crosstalk between tone and intonation in L2 acquisition. As will be shown below, this acquisition setting has hardly been studied. We use an imitation paradigm and test three mutually exclusive hypotheses. The first hypothesis (H1) states that Mandarin speakers produce the two kinds of contrasts similarly to native German speakers – given their increased sensitivity to pitch. The second hypothesis (H2) predicts a reduced ability to produce pitch accent contrasts in an L2 intonation language – given the documented sensitivity to tonal contrasts at the expense of processing phrase-level intonational contrasts. Finally, the third hypothesis (H3) predicts cross-linguistic influence (CLI), which refers to the transfer of native language features into the L2 (e.g., McManus, 2022). In particular, H3 predicts that pitch accent contrasts that are perceived as similar, possibly because they are mapped onto the same tones, are more difficult to imitate than pitch accent contrasts that are perceived as dissimilar.

Generally, L2 learners experience difficulties in the acquisition of a target-like intonation – both in perception and in production (e.g., Mennen, 2004, 2015; Liang and Heuven, 2009; He et al., 2012; Chen, 2014; Graham and Post, 2018; Trouvain and Braun, 2021; Shang and Elvira-García, 2022). In production, deviant intonation contours have been shown to lead to a perceived foreign accent (e.g., Willems, 1982; Anderson-Hsieh et al., 1992; Munro and Derwing, 1995; Magen, 1998; Jilka, 2000; Mennen, 2004; Trofimovich and Baker, 2006; Ulbrich and Mennen, 2016), lower intelligibility (Munro and Derwing, 1995; Holm, 2007), and may even slow down lexical processing (Braun et al., 2011). Although some L2 speakers sound more native-like than others, most L2 speakers still tend to show deviations in intonation patterns – even after having been exposed to their L2 for a long time (e.g., Mennen, 1998, 2004; Atterer and Ladd, 2004; O’Brien and Gut, 2010; Zahner and Yu, 2019; Manzoni-Luxenburger, 2021). Given that foreign accents may lead to reduced intelligibility (Munro and Derwing, 1999; Munro et al., 2006) and to negative attitudes toward the accented speakers (Munro et al., 2006), it is vital to understand the source of these difficulties. Acquiring L2 intonation is a complex endeavor since it involves the acquisition of different components on several linguistic levels. In particular, it requires the acquisition of three main components: (i) the phonological inventory of intonational events (i.e., a set of contrastive units – typically pitch accents and boundary tones), (ii) their phonetic implementation (e.g., tonal alignment), and (iii) their communicative function (semantics/pragmatics), *cf.* Mennen (2015). In the present study, we focus on the first two components.

Particularly in the domain of tone languages, only few studies have examined the acquisition of L2 intonation by L1 speakers of a tone language (He et al., 2012; Liu and Chen, 2016; Yuan et al., 2018; Liu and Reed, 2021; Shang and Elvira-García, 2022). These studies revealed effects of L2 proficiency and cross-linguistic differences, but very few studies have provided direct comparisons between learners whose L1 is a tone language versus learners whose L1 is a non-tone language.^1^ Also, prior studies often used tasks that required learners to access the semantic and pragmatic meaning, making it hard to determine genuinely phonetic and phonological factors. It is therefore unclear whether the lexical function of f0 puts learners at an advantage when acquiring L2 intonation, or, conversely, whether L2 intonation acquisition is made even more challenging. The present paper sets out to fill this gap by testing the crosstalk between tone and intonation in the acquisition of pitch accent contrasts by native speakers of a tone language.

In Experiment 1, we elicited L2 imitations of German pitch accent contrasts by speakers of Mandarin Chinese in two proficiency groups – and compared them to the native German productions analyzed in Zahner-Ritter et al. (2022). To gauge the difficulties in L2 acquisition for the two Mandarin proficiency groups and to study the role of L1 tone more directly, we included a control group of low-proficient Italian learners of German, whose L1 is an intonation language (Experiment 2). The paper is structured as follows. In the section “Background,” we first provide some background on the phonetics and phonology of pitch in German and Mandarin. Section “Experiment 1” presents the main experiment (Mandarin learners of German) and section “Experiment 2” describes the control experiment (Italian leaners of German). In the “General discussion,” we discuss CLI and crosstalk between tone and intonation in our data, as well as the role of proficiency, and end with a “Conclusion.”

## Background

Prosodic typology differentiates intonation languages and tone languages (Yip, 2002; Hyman, 2006).^2^ Broadly speaking, intonation languages use pitch movements to mark words that are prominent on the utterance level and at prosodic boundaries, while in tone languages, pitch movements and/or levels primarily mark lexical or grammatical meaning (Yip, 2002; Gussenhoven, 2004; Ladd, 2008). Since the present study tests the ability of speakers of a tone language to produce pitch accents in an intonation language, we briefly introduce general prosodic properties of intonation languages (“Phonology and phonetics of pitch accent contrasts in German”), with a focus on German rising-falling contours, the test case of this study, and tone languages, with a focus on Mandarin (“Tone and intonation in Mandarin Chinese”). In “Deriving hypotheses on the L2 acquisition of pitch accent contrasts,” we briefly survey the state of the art on the acquisition of pitch accents in an L2.

### Phonology and phonetics of pitch accent contrasts in German

In intonation languages such as German, English, or Italian, the speech melody comprises pitch accents, which are associated with metrically stressed syllables or prominent words, and boundary tones, which are associated with the edges of intonation phrases (Pierrehumbert, 1980; Beckman and Pierrehumbert, 1986; Ladd, 2008). Each utterance contains at least one intonation phrase and each intonation phrase, in turn, at least one pitch accent (Pierrehumbert, 1980; Nespor and Vogel, 1986). Pitch accents mainly signal post-lexical information, such as the discourse status of referents (given, new, accessible, *cf.* Baumann, 2006) the information structure of an utterance (focus vs. background, D’Imperio, 2001; Ladd, 2008, for overview), and speaker attitudes (Braun et al., 2019; Kutscheid and Braun, 2021; Wochner, Forthcoming); boundary tones mainly signal illocution types (question vs. statement, *cf.* Batliner, 1989; Oppenrieder, 1991; Grice, 1995; Niebuhr et al., 2010; Michalsky, 2017) and discourse organization (Lehiste, 1975; Wichmann et al., 2000).

**Figure 1:**
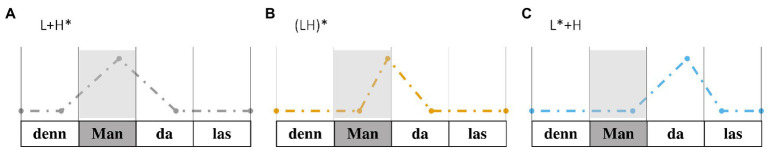
Schematic representation of three rising-falling contours in German realized on a four-syllable sequence *denn Mandalas* “PRT mandalas”; gray shading indicates the stressed syllable with which the pitch accent is associated. **(A–C)** show the three different alignment configurations analyzed in the present study.

The present paper focuses on the production of pitch accents. In autosegmental-metrical theory of intonation (Arvaniti and Fletcher, 2020 for overview; Pierrehumbert, 1980; Ladd, 2008), pitch accents are composed of low (L) or high (H) tonal targets (or a combination thereof). These tonal targets are associated with the metrically stressed syllable (e.g., the syllable [man] in <Mandalas> “mandalas”). Differences in the temporal alignment of the tonal targets with regard to the stressed syllable result in different pitch accent types. For instance, in an L + H* accent, the L tone precedes the stressed syllable, and the H tone is realized on the stressed syllable (symbolized by the asterisk), see Figure 1A. In contrast, an L* + H accent has its L tone aligned within the stressed syllable while the H tone is realized on the unstressed syllable following the stressed syllable, see Figure 1C. In the present study, we include a further accent type, termed (LH)*, which acoustically lies between the two and in which both L and H are aligned within the stressed syllable (Kohler, 2005; Zahner-Ritter et al., 2022), see Figure 1B.

A recent imitation study with German participants corroborated this three-way partition (Figures 1A–C) in imitated productions, in particular for speakers from Northern Germany (Zahner-Ritter et al., 2022). Pairwise comparisons of f0 values between these rising-falling contours revealed statistical differences in all cases, with a larger acoustic contrast between (LH)* vs. L* + H (orange contour, Figure 1B vs. blue contour, Figure 1C) compared to (LH)* vs. L + H* (orange contour Figure 1B, vs. gray contour, Figure 1A). The contours further elicit distinct interpretations in native speakers of German and are hence considered phonemic in the German pitch accent system (e.g., Kohler, 1991, 2005; Grice et al., 2005; Kügler and Gollrad, 2015; Lommel and Michalsky, 2017; Braun and Biezma, 2019; Zahner-Ritter et al., 2022). In *wh*-questions, L + H* and L* + H [gray (A) and blue (C) contours in Figure 1] were mostly associated with information-seeking meaning, while (LH)* [orange contour (B) in Figure 1] was interpreted as surprise, negative attitude, aversion, and rhetorical meaning (Zahner-Ritter et al., 2022). In declarative sentences, L + H* has been shown to signal new information, L* + H is associated with established facts, and (LH)* with surprise (Kohler, 1991, 2005; Baumann and Grice, 2006; Wochner, Forthcoming; Zahner-Ritter et al., 2022). Zahner-Ritter et al. (2022) directly compared the meaning attributions of these three accents in *wh*-questions and declarative sentences. While L + H* and L* + H were less distinct in meaning in questions, they were clearly differentiated in declaratives. Crucially, the “intermediate” (LH)* accent was distinct from the two other accent types in both sentence types, mostly being associated with surprise, aversion, or other attitudes. Given these differences in utterance meaning, learners of German eventually need to acquire this contrast in order to successfully communicate in their L2.

### Tone and intonation in Mandarin Chinese

**Figure 2:**
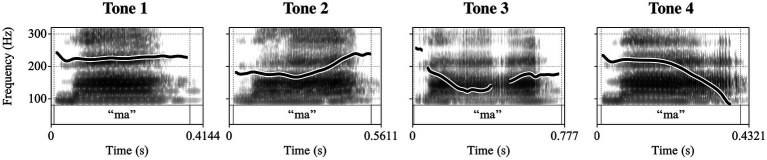
Example realization of lexical tones in Mandarin Chinese on the syllable “ma” (Tone 1 to Tone 4, from left to right), produced by a native speaker of Mandarin Chinese.

In tone languages, such as Mandarin Chinese, tones are used to differentiate lexical meanings. There are four lexical tones in Mandarin: Tone 1 which is high-level, Tone 2 which is high-rising, Tone 3 which is low-rising, Tone 4 which is falling (Chao, 1930, 1956; Lin, 2007, see Figure 2), and a neutral tone, which is prosodically weak and whose shape depends on the preceding tone (Cao, 1992; Yip, 2002; Zhang et al., 2022).^3^ Essentially, each syllable in a phrase carries one of these five tonal specifications, with the syllable boundaries generally serving as anchor points for the alignment of lexical tones (Xu, 1999). Lexical tones essentially determine the shape and height of the f0 contours within the syllable. They also influence the tonal configuration of the adjacent syllables, with tones in the preceding syllables showing stronger influence than the following one. In continuous speech, tones are coarticulated and reach their tonal targets late in the syllable as the f0 contour tends to show less influence and variation caused by the preceding tones toward the end of the syllable (Xu, 1999).

While f0 primarily marks lexical tone in Mandarin, it also conveys post-lexical meaning (Xu, 2019; Zhang et al., 2021, for overviews). The simultaneous existence of the lexical and post-lexical function of f0 (i.e., for tone and intonation) has been referred to as the “multiplexing of the f0 channel” (Zhang et al., 2021, p: 9).^4^ For instance, focus is marked by an increase in the f0 range on the focused word and by a compression of the f0 range in the post-focal region (Jin, 1996; Liu and Xu, 2005; Chen and Braun, 2006). Interrogatives are produced with higher overall f0 (Lee, 2005; Liu and Xu, 2005; Yuan, 2006), in particular towards the end of the utterance (Yuan, 2006). The tonal contour at the end of the utterance depends on the tone of the final constituent (Zhang et al., 2021 for overview). For instance, the falling Tone 4 is falling with a smaller range in questions as compared to statements; the rising Tone 2, on the other hand, is realized with an increased f0 range (Zhang et al., 2021) or higher register (Zhang et al., 2022). One approach to model and simulate the effects of lexical tone and intonation on the realization of the f0 contour in Mandarin Chinese is the *Parallel Encoding and Target Approximation model* (PENTA, Xu, 2005). In this model, each tone has an idealized pitch target, which may be static ([high], [low], [mid]), or dynamic ([rise], [fall]). The targets are approximated asymptotically, and, depending on the communicative function, the f0 range or the strength (speed) of target approximations is adjusted. In any case, this double-function of f0 leads to two processing consequences for Mandarin Chinese listeners: (1) an increased sensitivity to pitch (for tonal contrasts and musical pitch) and (2) a higher sensitivity to lexical tone, which is realized on syllabic units, than for intonation, which spans larger units. We now elaborate on (1) and (2) and formulate hypotheses.

### Deriving hypotheses on the L2 acquisition of pitch accent contrasts

The long-term experience with lexical tones leads to a higher sensitivity to musical pitch and improves general pitch processing (Wang et al., 2003; Wong et al., 2007; Zatorre and Gandour, 2008; Pfordresher and Brown, 2009; Bidelman et al., 2011, 2013; Bidelman and Chung, 2015). With regard to musical pitch, Pfordresher and Brown (2009), for instance, showed that L1 speakers of a tone language (Vietnamese, Mandarin Chinese, or Cantonese) outperform L1 speakers of an intonation language (English) on their ability to imitate (*via* singing) four-note sequences of different complexity (level, interval, melodic) and to differentiate between musical notes and musical intervals. The more complex the task, the larger the benefits. Furthermore, behavioral and neurophysiological evidence suggests differences in lexical tone and vowel identification (Gottfried and Suiter, 1997) and in the processing of level tones and contour tones between Chinese and English listeners (e.g., Gandour, 1983; Gandour et al., 2000). Crucially, long-term experience with lexical tones further leads to higher sensitivity toward pitch representations (Gandour et al., 1998, 2000; Krishnan et al., 2005, 2009, 2010; Krishnan and Gandour, 2009) and may enhance neuronal tuning of pitch in the brainstem (Gandour et al., 2000; Krishnan et al., 2009; Krishnan and Gandour, 2009), such that listeners are more accurate in detecting changes in pitch and musical intervals (e.g., interval distances and direction of change, Giuliano et al., 2011). Bidelman and Chung (2015) further showed that Mandarin Chinese listeners showed fine-grained distinctions of pitch encoding between hemispheres and differential processing of pitch contours and intervals, which was different from English listeners. There is also evidence from production supporting the idea that speakers of tone languages may be more sensitive to pitch cues than speakers of intonational languages. For instance, Keating and Kuo (2012) found that Mandarin speakers showed enhanced f0 profiles (higher maxima, larger ranges), especially for one-word utterances, compared to native speakers of American English. This increased sensitivity to pitch in general might therefore generate an advantage for speakers of a tone language when acquiring pitch accent categories in an (intonational) L2.^5^ These findings lead to **Hypothesis 1 (H1)** which states a **benefit of general pitch processing**, such that L1 speakers of a tone language are equally good at realizing L2 German pitch accent contrasts (see Figure 1) as native speakers and, crucially, better than learners of an intonation language (Experiment 2).

Meanwhile, several studies have documented that L1 speakers of a tone language are more sensitive to tone, typically restricted to the syllable, than to intonation, typically spanning larger domains (but see Ip and Cutler, 2017, 2020). Yuan (2011), for instance, showed that Mandarin Chinese listeners did not always reach high accuracy in identifying the correct illocution in their L1 (question vs. statement) based on intonational information alone. The lower accuracy occurred with specific lexical tones: Listeners were more accurate in identifying an utterance as a question when it ended in a falling tone (Tone 4, identification rate around 90%) as compared to when it ended in a rising tone (Tone 2, identification rate around 70%). This finding clearly reveals crosstalk between the two domains, see also Liu (2018). Liu et al. (2016a) further tested whether semantic context (neutral vs. providing sufficient information for the (tonal) identity of the final syllable) helped the identification of statement vs. question intonation. Even when the context was informative, identification results were comparable to Yuan (2011), with questions being easier to identify on falling tones. These behavioral findings on the crosstalk between tone and intonation are supported by electrophysiological evidence: Liu et al. (2016b) showed that Mandarin Chinese listeners distinguished between statements and questions based on intonation when the target sentence ended in Tone 4 (as evidenced by a P300 for questions relative to statements), but not when the target question ended in Tone 2 (where no ERP difference between questions and statements was found). This lack of sensitivity to phrase-level intonation also transfers to intonation processing in another tone language (Liang and Heuven, 2009) and to non-native processing (Braun and Johnson, 2011). In particular, Braun and Johnson (2011) used disyllabic nonce-words that had pitch movements resembling Tone 2 and Tone 4 on the first syllable in Experiment 1 or on the second syllable in Experiment 2. Chinese and Dutch listeners performed an ABX match-to-sample task with both sets of contrasts (between-subjects). They showed that Mandarin listeners were more attentive to pitch movements than Dutch listeners as these signaled potential lexical contrasts in Mandarin (but not in Dutch). Dutch listeners, in turn, were more attentive to pitch movements signaling post-lexical information than to pitch movements signaling no meaningful linguistic information. These findings lead to **Hypothesis 2** (H2, crosstalk between tone and intonation) which predicts that L1 speakers of a tone language have generally more difficulties in imitating L2 intonational pitch accent contrasts than learners of an intonation language (who are more used to pitch processing on domains larger than the syllable).

In the acquisition literature, there are only few studies on the acquisition of pitch accents. Trouvain and Braun (2021) recently summarized that most learner populations align low and high tonal targets differently from native speakers. Later alignment was shown for German learners of English (Atterer and Ladd, 2004; Gut, 2009; Ulbrich, 2013) as well as Japanese and Spanish low-proficient and high-proficient learners of American English (Graham and Post, 2018). Earlier alignment was reported for Dutch learners of Greek (Mennen, 2004) and Basque learners of Spanish, at least in the accents of object phrases (Elordieta, 2003). The fact that some learner groups align tonal targets later and other learner groups earlier suggests an influence of the respective L1. Learners not only deviate from the target in terms of the phonetic realization of pitch accents, but also in terms of the accent type that is used. Ramírez Verdugo (2006) reported that Spanish learners of English produced more rising accents on focused words than native English speakers, who, in turn, produced more falls. Mandarin Chinese learners of Spanish also tended to employ high/rising tunes to substitute Spanish low-pitched accents, along with a general tendency to compress pitch (Shang and Elvira-García, 2022). Most of these studies necessitate access to semantic/pragmatic information, beyond the actual realization of accentual contrasts, which obscures the source of the acquisition difficulties. In the present imitation paradigm, we directly access phonological acquisition. There is only one model on L2 intonation, the L2 Intonation Learning Theory (LILt, Mennen, 2015). In Mennen’s model, four aspects are argued to predict successful L2 intonation acquisition, (i) the inventory and distribution of phonological elements, (ii) the phonetic implementation of these elements, (iii) their function and (iv) their frequency of occurrence, hence connecting aspects of form and meaning/usage. Our imitation paradigm allows us to test (i) and (ii). The perceived (dis)similarity between Mandarin tones and the f0 contours on the three target syllables is of relevance for predicting the acquisition success. Native Mandarin Chinese listeners without prior knowledge of German reported that (LH)* and L + H* sounded similar to each other, while L* + H sounded clearly different. Hypothesis 3 (H3) states specific effects of CLI, such that (LH)* and L + H* are more difficult to acquire for L1 tone speakers as they are perceived as similar (**henceforth, “merger contrast”**), while (LH)* and L* + H, which are perceived as dissimilar (**henceforth, “non-merger contrast”**), are easier to acquire.^6^ Learners of another intonation language (e.g., Italian), will be exposed to different kinds of CLI and hence produce different intonational patterns.

In the LILt (Mennen, 2015), exposure is a relevant factor to predict successful acquisition of L2 intonation, and indeed empirical studies have shown that higher proficiency is beneficial in pitch accent acquisition (e.g., Baker, 2010; He et al., 2012; Graham and Post, 2018; Shang and Elvira-García, 2022). We therefore predict an effect of proficiency for all three factors described in H1-H3, but potentially in different directions: With respect to CLI (H3) and crosstalk between tone and intonation (H2), proficiency is expected to have a beneficial effect. **CLI** is expected to play a smaller role and the contrasts are produced more target-like (i.e., more similar to the native German realization of the contrast). **Crosstalk** might also be reduced such that learners with more experience of German are able to expand the processing window beyond the syllable, also leading to reduced interference of lexical tone specifics and hence to more target-like productions. In contrast, proficiency might have a reversed effect on the **general, non-linguistic pitch processing skills** in Mandarin learners (H1). Here, high-proficient learners might show a deeper (more linguistic) processing of the contours as compared to low-proficient learners, which might reduce the beneficial effect of general pitch processing advantages. Under this assumption, we predict more distinct contours for low-proficient than for high-proficient learners in production.

## Experiment 1

We tested Mandarin Chinese learners of German in two proficiency groups in a delayed imitation paradigm (see Zahner-Ritter et al., 2022, for use with native German speakers). Delayed imitation tasks are particularly suited for tapping into intonational development in phonology because no knowledge of semantics and pragmatics is necessary. In addition, the delay between stimulus and onset of imitation (here of 2.5 s) necessitates some kind of phonological storage, leaving little room for echoic (phonetic) memory (Baddeley, 1986, 2003). When speakers initiate their imitative productions after the delay, the phonetic trace has been decayed and speakers need to recruit phonological processing mechanisms. The paradigm hence directly assesses phonological processing and allows us to shed light on phonological acquisition processes in the L2 acquisition of pitch accent contrasts by L1 tone speakers.

For the analysis, we treat distinct f0 realizations at the group level as evidence for the formation of phonological categories. We processed the f0 contours of the imitations using General Additive Mixed Models (GAMMs, *cf.* Wood, 2006, 2017; Wieling, 2018; van Rij et al., 2019; Sóskuthy, 2021), which allow for a holistic comparison between *intonation condition* and *proficiency* and interactions between these factors over time and in comparison to native German speakers (data from Zahner-Ritter et al., 2022 is used for L1-comparisons). In intonation, there is always some variability, also among native speakers (*cf.* Zahner-Ritter et al., 2022), so we compared the learners’ productions to the whole group of native German participants to not disadvantage learners with less variable input. We focused on the realization of contrasts between pitch accents and tested how these contrasts differ between learners and native speakers. This allows us to reduce differences across participants (e.g., in f0 range) and to focus on the differences across pitch accents.

### Methods

#### Participants

Fifty-five native Mandarin speakers of L2 German participated in an online study *via SoSciSurvey* (Leiner, 2019). Participants filled in a meta-data questionnaire including self-rated proficiency based on the European reference framework ranging from A1 to C2 (Council of Europe, 2020). Proficiency was further measured using the lexical DIALANG test (Alderson, 2005).^7^ All participants confirmed to have at least beginner-level knowledge of L2 German (at least A1, otherwise the experiment ended automatically). The mean age of onset in German was 20.6 years (SD = 5.6). Participants participated from various locations in mainland China and Taiwan, with varying proficiency levels across regions. To avoid potential confounds between region and proficiency,^8^ we selected 28 participants (see Table 1) based on their proficiency and region of origin. The proficiency grouping was done based on the DIALANG score. Participants with values larger than 53 (i.e., more than 70% of the maximum number of points) were grouped as high-proficient, others as low-proficient. Low-proficient speakers most often indicated their German level as A2, high-proficient speakers as C1. In each proficiency group, 14 participants came from southern regions and 14 from northern regions. However, all of our participants indicated that Mandarin Chinese is their dominant language. Three of the high-proficient participants were living in Germany at the time of testing. None of the participants had any documented speech, hearing, or voice disorders.

**Table 1:** Overview of the Mandarin Chinese participants in Experiment 1. DIALANG scores range from 0 (no knowledge of German) to 75 (excellent knowledge of German). Foreign accent ratings range from 1 (no foreign accent) and 6 (strong foreign accent). For more details on the linguistic background see Supplementary material.

Proficiency	Age in years [mean and (sd)]	DIALANG score [mean and (sd)]	Foreign accent rating [mean and (sd)]
Low-proficiency group	22.4 (2.9)	45.4 (6.3)	4.9 (1.6)
High-proficiency group	23.7 (2.8)	59.9 (3.6)	4.0 (1.4)

After collecting all the data, we randomly selected two utterances from each participant. We asked 12 native speakers of German to rate these utterances for the strength of foreign accentedness on a scale from 1 (no perceivable foreign accent) to 6 (strong foreign accent), see Levi et al. (2007) or Hopp and Schmid (2013). The Mandarin utterances were interspersed with two utterances each from the Italian learners of German (reported in Experiment 2) and 16 utterances from German natives. Agreement among the 12 raters (Cronbach alpha, Cronbach, 1951) was very high (mean α = 0.97, 95% CI: [0.96; 0.98]). The last column of Table 1 shows the mean foreign-accent ratings, averaged across the 12 raters and the two recordings of each speaker. The German group had a mean accent rating of 1.0 (SD = 0.0). The difference in DIALANG scores was significant across proficiency groups (*t* = 7.73, *df* = 23.0, *p* < 0.0001), the same was true for mean foreign-accent ratings (*t* = −2.26, *df* = 53.9, *p* = 0.03).

#### Materials

We employed the stimuli from Zahner-Ritter et al. (2022), used in an imitation study with L1 German speakers. These were 4 *wh*-questions (e.g., *Wer malt denn Mandalas*, lit. “Who draws PRT mandalas?”), in which the final object noun had lexical stress on the first syllable (e.g., [man] in <Mandalas>). The *wh*-questions were recorded by a native speaker of German in two conditions (“source recordings”: L + H* and L* + H) and then resynthesized into three intonation conditions, all with a nuclear accent on the object noun: L + H*, (LH)*, and L* + H, with a final low boundary tone, see Figure 1. Differences in duration were removed by manipulating the stimuli such that each syllable had an average duration (within the four items *Mandalas* “mandalas,” *Malibu* “Malibu drink,” *Melanie* “Melanie,” *Libero* “libero soccer position”). The stimuli were further scaled in intensity to 63 dB. All manipulations were done in Praat (Boersma and Weenink, 2016), see Zahner-Ritter et al. (2022) for further details. In total, there were 24 test sentences (4 *wh*-questions × 3 intonation conditions × 2 source recordings).

#### Procedure

Participants first filled in a questionnaire before they performed the imitation task. After the imitation task they completed the lexical proficiency test (DIALANG). Participants were asked to prepare a computer (desktop or laptop) and headphones at the beginning of the experiment and were given explicit instructions [in German or Mandarin (self-chosen)] for the set-up of recording on their devices (e.g., browser settings). Participants were invited to take part in a lottery for reimbursement. All participants gave informed consent for participation and data processing. The study was conducted remotely *via SoSciSurvey* (Leiner, 2019), ran on an in-house server.

For the actual imitation task, participants were randomly assigned to one of two experimental lists (differing in order of items to avoid position effects). The stimuli were played once followed by 2000 ms silence and a 500 ms sine tone (randomly played at 150 Hz or 450 Hz) to reduce the impact of purely phonetic processing (Plomp, 1964; Baddeley and Hitch, 1974). The experiment started with four practice trials to familiarize participants with the voice and the task. Participants were instructed to imitate the target contours as closely as possible. They were additionally advised to complete the study in one go in a quiet environment to minimize background noise and interference during the recording. The recording process began automatically after the second sine tone and ended when participants clicked a key to move on to the next page. Participants were allowed to repeat themselves in case of mistakes or when they were dissatisfied with the recording. In that case, we analyzed the final production. In terms of variables, *intonation contour* was manipulated within-subjects and within-items, so that each participant imitated 24 *wh*-questions overall (4 *wh*-questions × 3 intonation conditions × 2 source recordings).^9^

#### Data treatment

**Figure 3:**
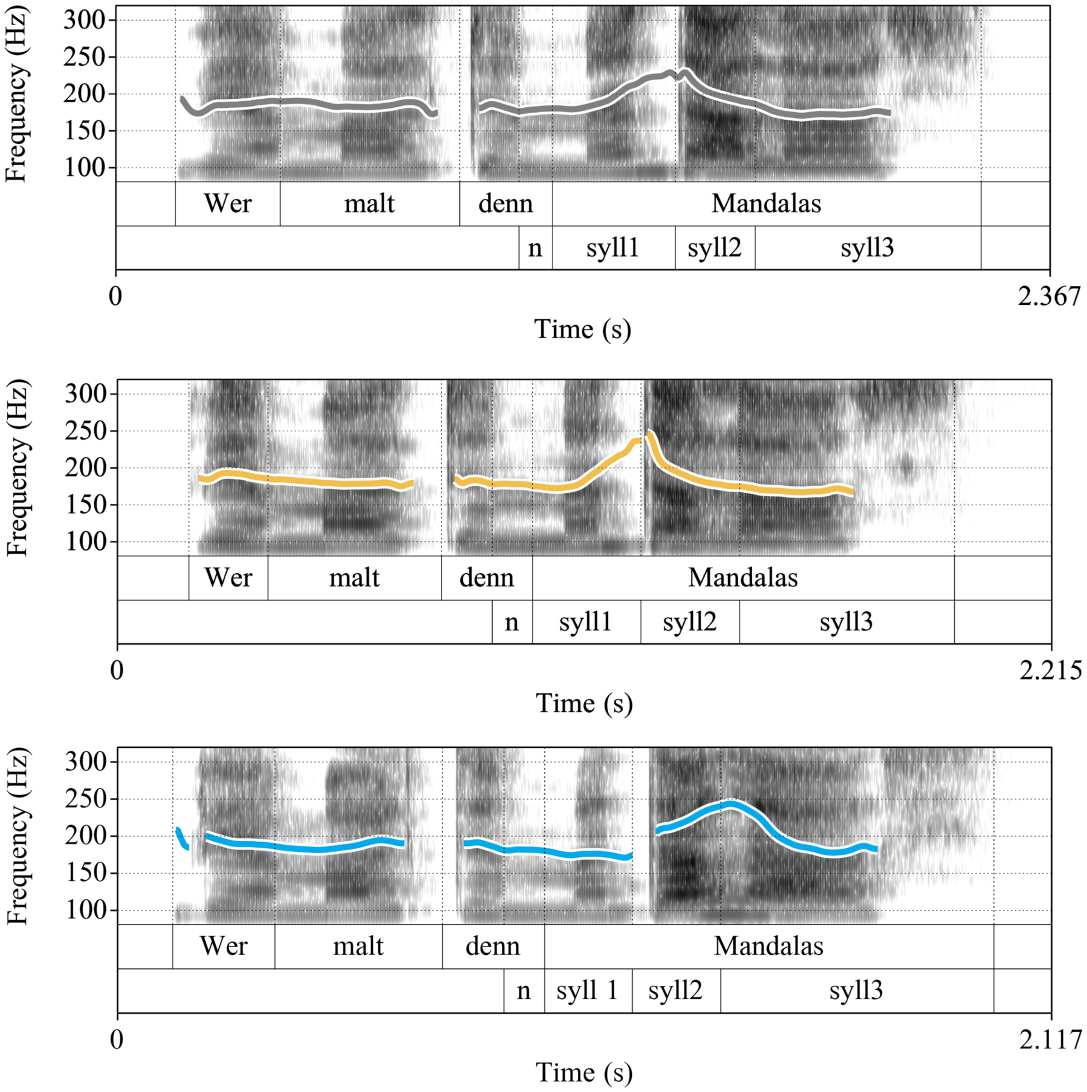
Imitative productions of the target question *Wer malt denn Mandalas?* (“Who draws mandalas?”) in the three intonation conditions (vp07, Mandarin Chinese low-proficiency group, female, 29 years). Top panel: L + H*, mid panel: (LH)*, bottom panel: L* + H. The filled intervals from tier 2 served as input tier for the extraction of f0 values.

The sound files were annotated semi-automatically: The initial segmentation generated by *Web-MAUS* (Kisler et al., 2017) was corrected manually where necessary according to standard segmentation criteria, *cf.* Turk et al. (2006), see Figure 3 for analysis tiers and exemplar realizations in the three conditions. The annotation and analysis focused on the final segment [n] of the particle *denn* and the three syllables in the sentence-final noun (e.g., *Mandalas*), as the study concentrated on the production of nuclear pitch accents (see Tier 2 in Figure 3).

**Figure 4:**
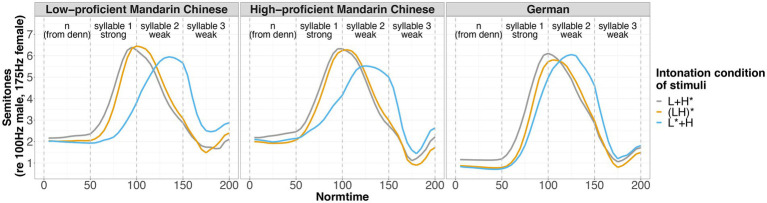
Average f0 contours (in st) in the three intonation conditions, split by proficiency group. Left panel shows low-proficient learners of German, middle panel high-proficient learners of German, and right panel German native speakers from Zahner-Ritter et al. (2022).

F0 values were extracted using ProsodyPro (Xu, 2013) with 50 measurements per syllable. This was done separately for male (*N* = 1) and female speakers (*N* = 27), with different extraction settings for f0-minima and maxima (male: 50–300 Hz; female: 100–500 Hz). The raw f0 values were down-sampled to 10 values per interval for subsequent statistical analyses and converted to semitones (reference level was set to 100 Hz for male and 175 Hz for female speakers). Figure 4 shows the average f0 contours of the low- and high-proficient Mandarin learners of German along with the German native speakers (Zahner-Ritter et al., 2022).

We merged the German dataset reported in Zahner-Ritter et al. (2022), see right panel in Figure 4, with the Mandarin Chinese dataset (middle and left panel in Figure 4) and coded three language-groups: *low-proficient Mandarin Chinese learners*, *high-proficient Mandarin Chinese learners*, and *German native speakers*. We then used General Additive Mixed Models (GAMMs, Wood, 2006, 2017) to test whether the three groups differ in the realization of the three intonation conditions over time. GAMMs allow for a direct comparison between f0 contours because they can model non-linear dependencies of a response variable (here f0 in semitones) and different predictors (here *intonation condition* and *group* and their interaction) over time *via* smooth functions. They do so by using a pre-specified number of base functions of different shapes (Baayen et al., 2018; Wieling, 2018; van Rij et al., 2019; Sóskuthy, 2021). Such direct comparisons between f0 contours allow us to study the realization of accentual contrasts in different speaker groups. Of particular importance are differences in f0 over time in the realization for two kinds of pitch accent contrasts:

- **Non-merger contrast**: (LH)* vs. L* + H (orange vs. blue contour in Figure 1)
- **Merger contrast**: (LH)* vs. L + H* (orange vs. gray contour in Figure 1)

The dependent variable was the f0 value [in semitones (st)]. Models were initially fitted using the maximum likelihood (ML) estimation method in order to be able to compare models with different complexity (Sóskuthy, 2021, p: 16; Wieling, 2018, p: 89). This allowed us to test whether the interaction significantly improved the fit of the model, compared to a model without an interaction term. Since autocorrelation between values of a variable is problematic and since f0 values at subsequent timepoints are necessarily correlated, we corrected for this by using an autocorrelation parameter *rho*, determined by the acf_resid() function in the package *itsadug* (van Rij et al., 2017). We modeled separate smooths for subjects and items to account for the experimental structure. Model fits were finally checked using gam.check() and the number of base functions (k) was adjusted if necessary. Also, models were re-run with the scaled *t* distribution (family = “scat”), closely following the suggestion in van Rij et al. (2019, p: 17) to account for tailed residuals. For the model fitting of the GAMMs, we used the R package *mgcv* (Wood, 2011, 2017); the package *itsadug* was used to plot the model results (van Rij et al., 2017). Given that the interpretation of significant differences is only possible through visualization, we present the visualized model output. The steps of the analyses are available on Mendeley http://doi.org/10.17632/w293n86sjr.2.

### Results and discussion

The model with the smooth term for the interaction between *condition* and *group* over time was significantly better than the model without this interaction [*𝜒^2^*(18.00) = 271.240, *p* < 2e−16], suggesting that the groups differ in the realization of the f0 contours. The final model (with the scat-linking function), corrected for autocorrelation, accounted for 68.6% of the variance.

#### Non-merger-contrast: (LH)* vs. L* + H

**Figure 5:**
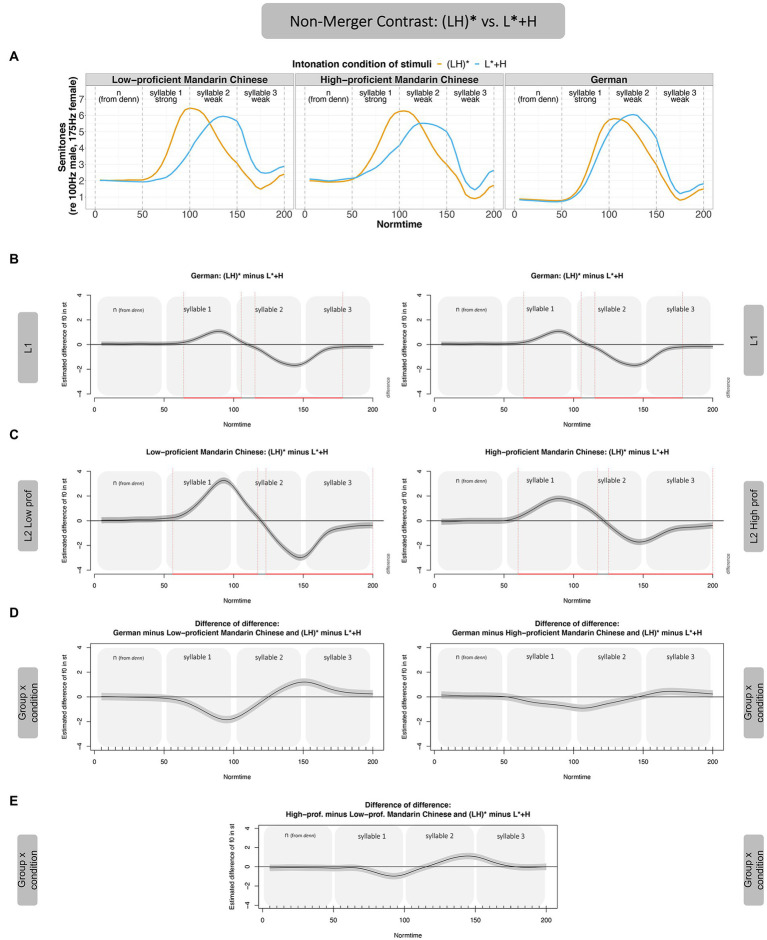
GAMM results—Non-Merger Contrast, (LH)* vs. L* + H, by Mandarin learners. Panel **A** shows the contours of the non-merger contrast across participant groups. Panel **B** shows the realization of the contrast in form of difference curves [(LH)* minus L* + H] over time for L1 German (duplicated to make later comparison with the two proficiency groups more transparent, i.e., same figure on left and right). Panel **C** shows the difference curves for the two proficiency groups, Mandarin low-proficient (left) and high-proficient learners (right). Panel **D** shows the difference of the difference between L1 and L2 in the non-merger contrast (i.e., the interaction between condition x group), Panel **E** the difference of the difference between the two proficiency groups.

We start with the distinction of the non-merger contrast [(LH)* vs. L* + H], see Figure 5 for an overview of results. Figure 5 (Panel A) shows the realization of the non-merger contrast in the different groups. Differences between f0 contours can be assessed in GAMMs with so-called difference curves where one contour is subtracted from the other. In Figure 5 (Panel B), the f0 values of L* + H (blue contour) are subtracted from the f0 values in (LH)* (orange contour). This procedure reveals when in time two f0 contours significantly differ from each other (in case zero is not included in the gray 95% Confidence Interval (CI), indicated by red vertical lines). In terms of L2 acquisition, we interpret distinct f0 contours as evidence for successful category formation. Figure 5 first shows the difference curve for the German L1 data from Zahner-Ritter et al. (2022) in Panel B; Panel C presents the difference curves for the Mandarin Chinese learners of German (low-proficient speakers on the left and high-proficient speakers on the right). Panel D finally presents the difference of the two difference curves shown in B (German) and C (learner groups), hence representing the interaction between *intonation condition* and *group*.

Plotted in terms of such a difference curve (Panel B), the (LH)* contour in **German native speakers** has higher f0 values than the L* + H contour in the stressed syllable (positive difference), and, conversely, the (LH)* contour is lower than the L* + H contour in the post-stressed syllables (negative difference). These differences augment to an absolute value of around 1 st in the stressed and to 2 st in the post-stressed syllable. Also, the L1 German speakers differentiate between (LH)* and L* + H mostly in terms of f0 peak alignment (H tone). The f0 peak occurs late in the stressed syllable of the noun for (LH)* and in the post-stressed syllable for L* + H. Both **learner groups** [Panel C, Mandarin low-proficient learners (left) and Mandarin high-proficient learners (right)] show the same general pattern, but, crucially, tend to make the difference between the two accents acoustically more extreme as compared to the German native speakers (as shown by a larger excursion of the difference curves on the y-axis, compared to the German speakers in Panel B). The contours (LH)* and L* + H are hence further apart in learners than in native speakers. Also, contours in Mandarin Chinese learners start to diverge slightly earlier in the stressed syllable (around Normtime 60) as compared to German native speakers (around Normtime 70), especially for the low-proficiency group. Panel D shows the difference of these differences to pin down group comparisons (German native speakers vs. the two learner groups). To this end, we subtracted the f0 values in the Mandarin groups (low-proficient on the left, high-proficient on the right) from the German group. Since the Mandarin groups show larger f0 differences in the stressed syllable (Normtime 50–100) than the German group, the difference of the difference in Panel D is negative. In the post-stressed syllable (Normtime 100–150), the larger difference of the Mandarin groups reverses. The data hence reveal that learners realize the merger contrast differently from the German native speaker group (more extreme). However, there is also a clear effect of proficiency: The high-proficient Mandarin learners are closer to the German speakers (closer to 0, whereby 0 indicates no deviation from the target) than the low-proficient Mandarin learners, a difference which is significant (see Panel E, which directly compares the contrast in the two learner groups).

Taken together, both high- and low-proficient Mandarin learners produced the pitch accent contrasts in an acoustically more pronounced way than German native speakers. These findings suggest that the perceived difference between the accents (L* + H was judged as different from the other two accents) is clearly measurable in a production experiment, which does not demand conscious judgment. Furthermore, the data show that the effect of perceived (dis)similarity has less effect on high-proficient learners, with high-proficient learners being on average closer to the target than the low-proficient learners. With regard to the realization of the accent types, Mandarin learners realized the rise considerably later in the L* + H accent (compared to the German natives). It is possible that the “late” peak (which was aligned in the post-stressed syllable) was parsed as a tone on the post-stressed syllable, which led to realizations that differed from those of native German speakers.

#### Merger-contrast: (LH)* vs. L + H*

**Figure 6:**
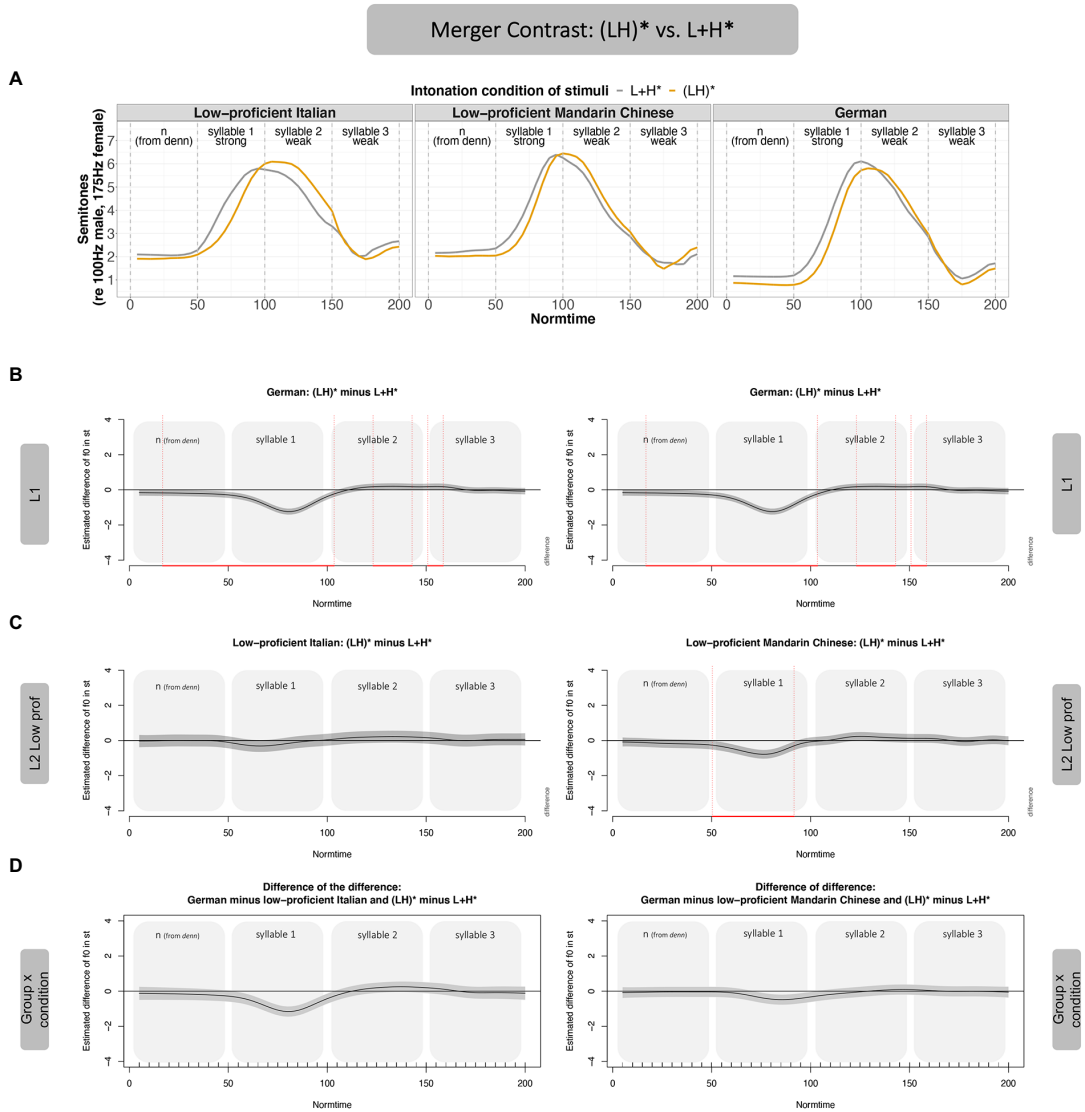
GAMM results—Merger Contrast, (LH)* vs. L + H*, by Mandarin learners. Panel **A** shows the contours of the merger contrast across participant groups. Panel **B** shows the realization of the contrast in form of difference curves [(LH)* minus L + H*] over time for L1 German (duplicated to make later comparison with the two proficiency groups more transparent, i.e., same figure on left and right). Panel **C** shows the difference curves for the two proficiency groups, Mandarin low-proficient (left) and high-proficient learners (right). Panel **D** shows the difference of the difference between L1 and L2 in the merger contrast (i.e., the interaction between intonation condition and group).

Figure 6 (Panel A) shows that the merger contrast is acoustically less pronounced than the non-merger contrast across the board (both in native speakers and the two learner groups). In analogy to Figure 5, the f0 values of L + H* (gray contour) are subtracted from the f0 values in (LH)* to arrive at the difference curves (Panels B and C). The difference curves in Panel B show that L1 German speakers differentiate between (LH)* and L + H* such that (LH)* has lower f0 values than L + H*, leading to a negative f0 difference. In the last two thirds of the post-stressed syllable, the (LH)* contour has slightly higher f0 values than L + H*, leading to a positive shift in the difference curve. The two Mandarin Chinese proficiency groups show largely the same pattern as the German native speakers (Panel C), leading to very minor differences of the difference for both speaker groups (Panel D). If anything, the low-proficiency group approached the German native speakers’ realization of the contrast more closely than the high-proficient group, evidenced by smaller deviations from 0 in Panel D (left). The low-proficient group, however, showed the differences in the stressed syllable only (i.e., in a smaller time interval than the German native speakers). The accentual differences of the high-proficient learners, in turn, were distributed in the same time intervals as the German native speakers’ contrast, but the contrast was smaller for high-proficient leaners than for the German native speakers. The differences between the proficiency groups were numeric only; the interaction between group and proficiency was not significant and is therefore not shown in Figure 6.

Taken together, for the comparison between (LH)* and L + H* (merger contrast), in which the f0 peak (H) was realized in the stressed syllable in both accents, German speakers realized the f0 difference mostly on the stressed syllable (Normtime 50–100), with a slight difference already on the pre-stressed syllable. There were differences in the post-stressed syllable, but these were small. The two learner groups showed a similar pattern, but the difference between the two contours was smaller than in native speakers. For the merger contrast, there was no effect of proficiency.

#### Interim discussion

Summarizing the results of Experiment 1, the non-merger contrast [i.e., (LH)* vs. L* + H] was produced more distinctly by the Mandarin Chinese learners compared to the German native speakers. Auditory impressions by native German speakers even suggested that some of the L* + H realizations in the Mandarin group led to a stress shift, such that the second, unstressed syllable of the noun sounded stressed (instead of the intended first syllable). This perception is most likely driven by the shallower slope of the rise in the stressed syllable, but this needs further investigation. In any case, the larger acoustic contrast in the non-merger contrast by the Mandarin learners (in particular the low-proficient ones) is not target-like. Proficiency seemed to boost the acquisition of this contrast. That is, the high-proficient learners were closer to the native speakers, suggesting that increased experience with an intonation language helps to reduce transfer from the L1. In the merger contrast [i.e., (LH)* vs. L + H*], both learner groups were significantly less distinct than the German native speakers, with no statistical effect of proficiency.^10^

The present data thus reveal an **asymmetrical pattern of pitch accent-contrast acquisition**. Mandarin learners of German are more distinct than German L1 speakers in the non-merger contrast and less distinct in the merger contrast. Such different acquisition outcomes for the two kinds of contrasts had indeed been hypothesized by H3, which based its predictions on CLI. We will return to the discussion of CLI in more detail in the “General Discussion.” The imitation data are not in line with H1 (general pitch processing benefit) or H2 (crosstalk), which predicted either **general** benefits or disadvantages for speakers of a tone language in pitch accent processing, i.e., a similar behavior for both kinds of contrasts. With respect to proficiency, our data partly support what has been predicted, since higher proficiency led to more target-like realizations, at least in the non-merger contrast. In the merger contrast, however, the influence of the L1 seems to override effects of proficiency, such that both learner groups produce the contrast in the same way. In Experiment 2, we test whether this pattern of CLI is specific to Mandarin learners or may also be observed in learners whose L1 is an intonation language. In a strong interpretation of H3, L1 speakers of an intonation language (Italian) will produce the contrasts differently than the L1 speakers of a tone language in Experiment 1. These data from learners of a non-tonal language will help us to interpret the type of CLI observed in Experiment 1 better.

## Experiment 2

In this control study, we tested a group of low-proficient Italian learners of German using the same paradigm as in Experiment 1. Like German, Italian is an intonation language which highlights words by means of pitch accents (Grice et al., 2001; D’Imperio, 2002; Gili Fivela et al., 2015).^11^ Importantly, Italian has a different set of pitch accents and phonetic realizations of these accents than German. It is hence well suited to act as control condition for the performance of the L1 speakers of a tone language who acquire an intonation language (see Experiment 1). If the differences in the realization of the accentual contrasts between the Mandarin learners of German and the German natives is indeed caused by CLI [i.e., that Mandarin participants perceive (LH)* and L + H* as similar, but L* + H as distinct from the two], we expect the Italian learners to produce contrasts closer to the German target (and hence more distinct from the Mandarin learners). If the two learner groups (Mandarin vs. Italian) do not differ, the underlying cause may also be a language-independent psychoacoustic processing mechanism or specific properties of the stimuli.

Note that we keep the terms “non-merger contrast” for (LH)* vs. L* + H and “merger contrast” for (LH)* vs. L + H* also for Italian participants – even though they were established based on the perception of (dis)similarity by Mandarin listeners, since this makes comparison to the Mandarin data easier.

### Methods

We used the same online imitation experiment as in Experiment 1, but tested a group of L1 Italian speakers with low proficiency in L2 German.

#### Participants

We recruited eight low-proficient Italian learners of German (6 female, 2 male; mean age: 29 years, SD: 9.25). They were from the North/Centre of Italy (region of birth: Piedmont: one speaker, Lombardy: four speakers, Veneto: one speaker, Trentino: one speaker, Tuscany: one speaker). One of them lived in Germany and one lived in the US at the time of testing. On average, the participants studied German for 2.9 years (SD: 1.8). Regarding self-rated proficiency based on the European reference framework (Council of Europe, 2020), they most often indicated their level as B1 (A1: two speakers, A2: one speaker, B1: four speakers, B2: one speaker). The Italian low-proficiency group had a mean DIALANG score of 46.3 (SD = 5.4); the score did not differ from the score of the low-proficient Mandarin speakers (45.3, *p* > 0.7). The mean foreign accent rating was 3.9 (SD = 1.5) and did not differ from the Mandarin Chinese participants’ rating either (*p* > 0.2).

#### Materials, procedure and data treatment

The materials, procedure and data treatment were the same as in Experiment 1, except that the segmentation of the four critical intervals ([n] from *denn,* and the three syllables of the sentence-final object) was done manually instead of using *WebMAUS* for an initial segmentation. Six imitations had to be excluded from the analyses due to background noise, hesitations, pauses, or lexical mistakes.

### Results and discussion

The data were processed and analyzed as in Experiment 1.

#### Non-merger contrast: (LH)* vs. L* + H

**Figure 7:**
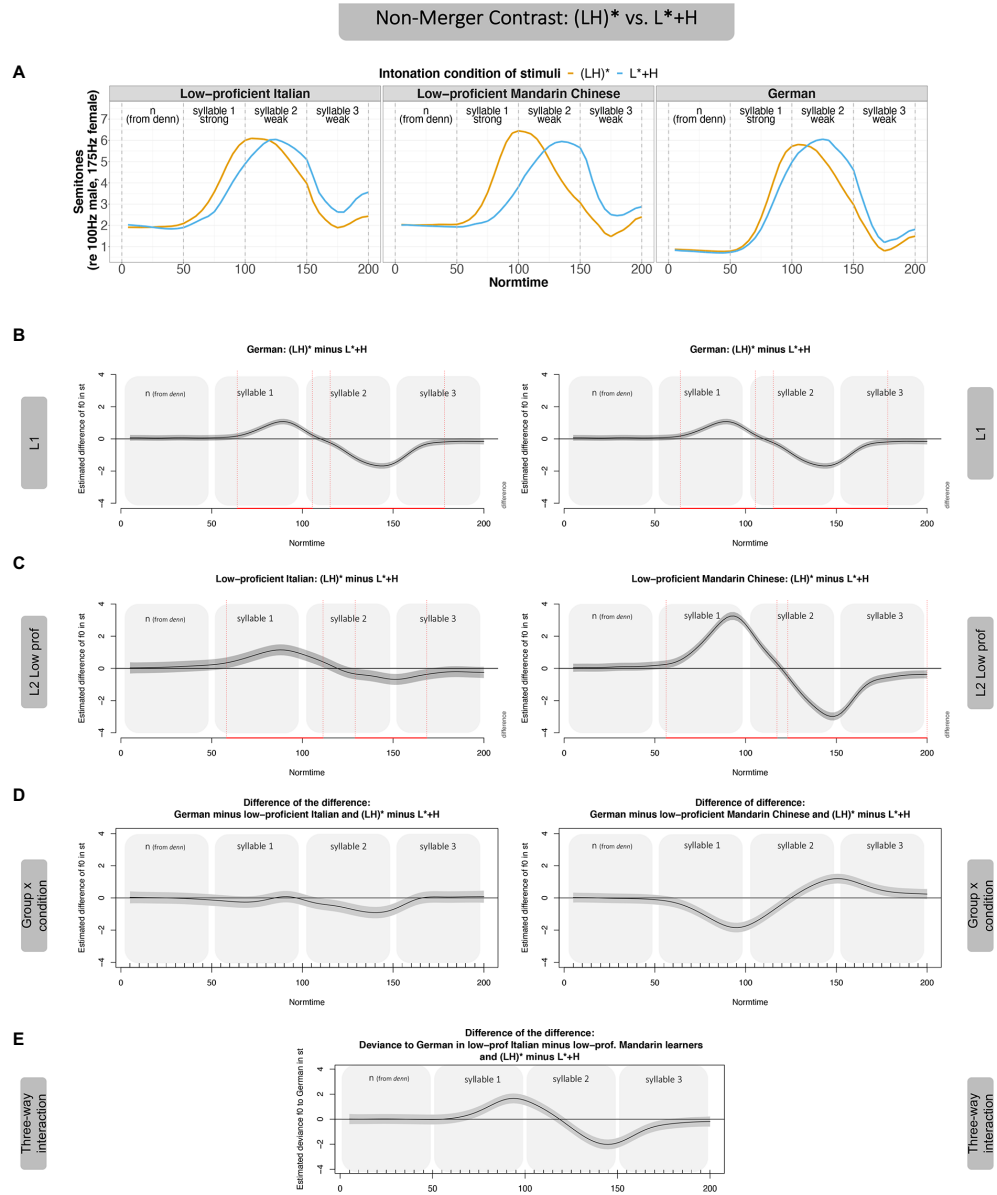
GAMM results—Non-Merger Contrast, (LH)* vs. L* + H, by Italian learners. Panel **A** shows the realization of the two contours of the non-merger contrast across participant groups. Panel **B** shows the difference curves for German, Panel **C** for the two low-proficient learner groups (Italian left, Mandarin Chinese repeated right). Panel **D** shows the difference of the difference, directly comparing the realization of the contrast compared to German native speakers. Panel **E** shows a direct comparison of the difference of the L2 groups compared to German.

We first analyzed the data in analogy to the Mandarin Chinese data. For direct comparison between learner groups, we display the low-proficient Italian data in the left panel of the figures and the low-proficient Mandarin data in the right panel (Panel C and D, Figure 7). We first combined the Italian data with the German data and tested whether a model with a smooth term for the interaction between *language* and *intonation condition* over time was better than a model with a condition-smooth only, which was the case [*𝜒^2^*(9.00) = 20.011, *p* < 0.001]. This final model (with the scat-linking function) explained 68.0% of the variance.

Figure 7 shows the two accent conditions of the non-merger contrast (Panel A), followed by difference curves for German (Panel B) and the two learner groups (Panel C). The Italian learners’ realizations of the contrast are closer to the German native speakers’ than the Mandarin learners’ realizations (even though the contours also start to diverge a little earlier than for German native speakers).^12^ This difference between learner groups is supported by the difference of the difference plots, which show differences between the Italian and German realization of the contrast (Panel D). These plots (Panel D) also reveal that both groups deviate from German native speakers (both deviate from 0). The accentual realization of Italian learners mostly differed in the post-stressed syllable from the German native speakers, but overall, the contrast was acoustically reduced. As will be discussed in the “General Discussion,” this temporal interval for the deviance may potentially be explained by the Italian accentual system, lending further support to CLI (H3).

The descriptive difference in the realization of the contrast between Mandarin Chinese and Italian learners (Panel C and D, left and right) is statistically corroborated as follows: We generated a derived dependent variable that captures the deviance of a learner from the average German speaker. To this end, we averaged the f0 values of the German speakers for each time point and subtracted this value from the learners’ f0 values over time. We then run the GAMM with this derived dependent variable, testing whether an interaction term for *condition* and *learner group* is significant. Model comparisons showed that the model with the interaction was significantly better than the model without the interaction term [*𝜒^2^*(9.00) = 118.180, *p* < 2e−16]; it accounted for 64.6% of the variance. The difference between learner groups in the deviance from German native speakers is directly shown in Panel E. Since the realization of the contrast in the post-stressed syllable is opposite in the two learner groups, the difference between these two groups is aggravated in this time interval.

#### Merger contrast: (LH)* vs. L + H*

**Figure 8:**
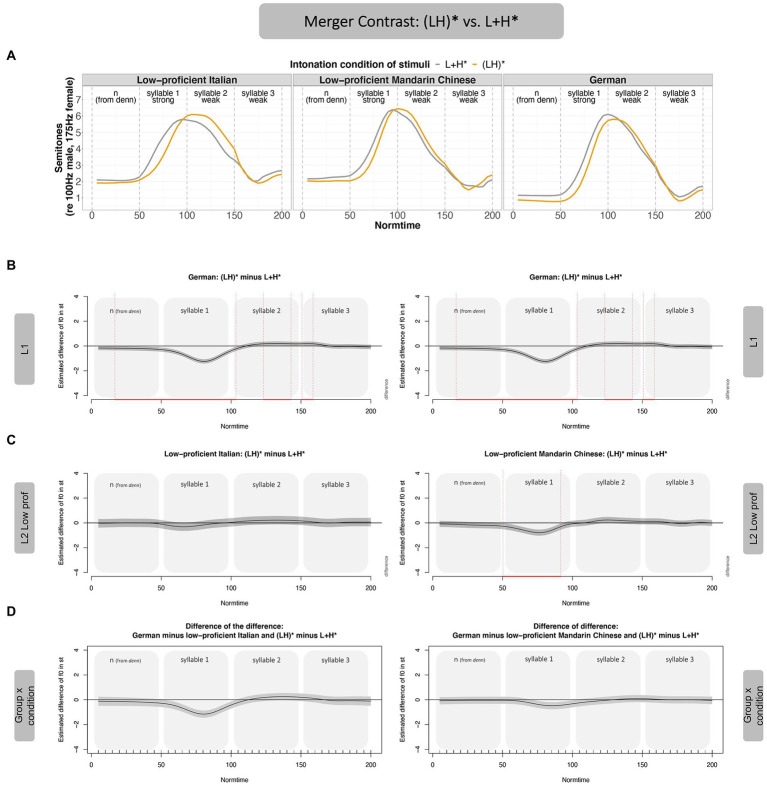
GAMM results—Merger Contrast, (LH)* vs. L + H*, by Italian learners. Panel **A** shows the realization of the two contours of the merger contrast across participant groups. Panel **B** shows the difference curves for German, Panel **C** for the two low-proficient learner groups (Italian left, Chinese repeated right). Panel **D** shows the difference of the difference, directly comparing the realization of the contrast compared to German native speakers.

The realization of the contrast between (LH)* and L + H* is shown in Figure 8. Italian learners did not realize the contrast but merged the two contours (Panel C left), leading to a significant difference compared to the German native speakers (Panel D left). Recall that the Mandarin learners realized this contrast (Panel C right), but less distinctly than the German native speakers. A direct comparison of the realization of this contrast across L1s (Mandarin Chinese vs. Italian) revealed that the interaction between *language* and *condition* was not significant (and is therefore not shown). Hence, there is no evidence to postulate differences in the realization of the contrast across learner groups (Italian vs. Mandarin Chinese).

The low-proficient Italian learners of German realized both contrasts less distinctly than the German native speakers. The non-merger contrast [(LH)* vs. L* + H] resulted in a significant difference across learner groups (with Mandarin Chinese learners deviating more from the German native speakers than the Italian learners). Given that the proficiency was largely matched across groups, the difference in imitation is likely due to the prosodic system in the native language (tone language vs. intonation language). For the merger contrast, learner groups did not significantly differ from each other; both realized the contrast significantly less distinctly than German native speakers. Note that the average contours of the accents [(LH)* vs. L + H*] for Italian speakers (Panel A) might suggest a difference, but there was great variance (broader confidence intervals in Panel C) and a small number of learners (eight Italian learners as compared to 14 Mandarin learners) – factors that may have prevented this descriptive difference to reach statistical significance.

## General discussion

The present study addressed the possibility of crosstalk between tone and intonation by studying the L2 acquisition of pitch accents [German L + H*, (LH)*, and L* + H] by Mandarin Chinese learners of German. Introspective judgements by Mandarin Chinese L1 speakers had suggested that (LH)* and L + H* may be prone to a merger effect because they are perceived as similar, and clearly different from L* + H. We hence based our predictions and analyses on two kinds of pitch accent contrasts, both involving a comparison to the acoustically intermediate condition, i.e., to the (LH)* accent: (1) a “non-merger contrast,” (LH)* vs. L* + H, and (2) a “merger contrast,” (LH)* vs. L + H*. Based on the literature, we formulated three hypotheses for the realization of these two pitch accent contrasts by L2 speakers. The first two are general hypotheses that are based on the fact that Mandarin is a tone language, the third hypothesis is based on CLI of lexical tone on pitch accents in the L2. H1 stated that Mandarin Chinese learners are equally good in imitating the two pitch accent contrasts as German native speakers because of an enhanced sensitivity to pitch in general (i.e., same pattern for both non-merger and merger contrast) with no effect of proficiency. Our data clearly falsified H1. The data also falsified H2, which stated a general disadvantage for acquiring intonational pitch accents for Mandarin Chinese learners. However, our data are partly compatible with H3, which stated that Mandarin Chinese learners produce the non-merger contrast [(LH*) vs. L* + H] equally distinct as German natives or even more pronounced, and the merger contrast [(LH)* vs. L + H*] less distinct compared to German natives due to CLI. Our findings support Mennen’s L2 intonation model (Mennen, 2015) in showing that the perceptual (dis)similarities are a relevant factor for the successful acquisition of pitch accent contrasts. Increased proficiency was claimed to reduce the effect of CLI in the L2 productions, which was the case for the non-merger contrast (but not for the merger contrast). In terms of proficiency, our findings only partly support Mennen’s LILt (Mennen, 2015). Experiment 2, a control experiment with native speakers of an intonation language (Italian), corroborated the effects of CLI: Their productions of both contrasts were closer to the German speakers’ productions than the productions by L1 Mandarin learners, in particular in the non-merger contrast condition.

Given the comparatively few studies on the phonological acquisition of pitch accents to date, it is difficult to devise models on this kind of CLI at this point. Clearly, more research from other typologically different L1s is necessary to corroborate the cross-linguistic differences and to disentangle the specifics of the L1 influence. In future research, we also plan to complement the imitation data by perceptual tasks (same-difference task) to locate the source of the CLI (in perception or in production). Moreover, it will be useful to test the more general hypotheses H1 and H2 with populations that have no or only very little experience with intonation languages (e.g., school pupils) to minimize effects of exposure. For such an endeavor the delayed imitation paradigm may be too challenging, though. Instead, a simplified version of the task, as has been used in other studies (e.g., an immediate imitation paradigm with multiple exposure to the target utterances, D’Imperio et al., 2014; Zahner-Ritter et al., 2021; Zhao, 2022), may be better suited because it allows participants to directly access the acoustic trace. Another way to simplify the task would be to use shorter utterances (only the object noun), and/or reiterant speech (Larkey, 1983; Rietveld et al., 2004).

In the remainder of this section, we reflect on the nature of CLI and the crosstalk between intonation and lexical tone in our data (“Crosstalk between tone and intonation and cross-linguistic influence”) before we briefly turn to the effect of proficiency (“Proficiency”).

### Crosstalk between tone and intonation and cross-linguistic influence

The type of crosstalk we observe between tone and intonation in L2 acquisition is one of general nature and difficult to disentangle from CLI as our results do not fully support H3. Tone language learners of an intonation language did not generally profit from their enhanced pitch processing abilities shown in other domains (crosstalk, see Wang et al., 2003; Wong et al., 2007; Zatorre and Gandour, 2008; Pfordresher and Brown, 2009; Bidelman et al., 2011, 2013; Bidelman and Chung, 2015). Otherwise, we would have expected target-like realizations of the contrasts, which was not the case. Also, the documented difficulty in intonational processing in their L1, in tonal L2s, and in non-native speech did not transfer to the acquisition of German pitch accents. If this had been the case, we would have expected a poor realization of the contrast across the board. Rather, we observed a nuanced (and asymmetrical) pattern in which the non-merger contrast [(LH)* vs. L* + H] was more distinct in Mandarin Chinese learners compared to the realizations of the German native speakers, while the merger contrast [(LH)* vs. L + H*] was less distinct compared to German native speakers. The merger contrast was closer to the target than the non-merger contrast, which, in turn, was clearly exaggerated. The large distinction of contours of the Mandarin participants for the non-merger contrast [(LH)* vs. L* + H] is likely due to the fact that the L* + H pitch accent was perceived differently from the other two accents, with the late peak (on the post-stressed syllable) being salient for listeners. This increased prominence on the post-stressed syllable for the L* + H might have hindered learners to perceive this contour as a pitch accent associated with the stressed syllable, followed by unstressed syllables, but at times as a pitch accent associated with the post-stressed syllable (*cf.* Kutscheid et al., 2021). Interestingly, some Mandarin productions of L* + H (in particular in the low-proficient group) sounded as if they were stressed on the post-stressed syllable (i.e., resulting in the perception of primary stress on the second syllable, as judged by German native speakers). Hence, crosstalk in our study becomes evident in that learners with a tone language as L1, in particular the low-proficient ones, seem to be influenced by their tonal phonology when processing pitch accents in the L2.

The fact that Mandarin Chinese learners were not generally disadvantaged in imitating pitch accent contrasts (contra H2) may have different explanations. Conceivably, the decreased sensitivity to pitch was mostly documented for the question-statement contrast toward the end of the utterance, while the contrast we tested was a pitch accent contrast in the middle of the utterance. Ip and Cutler (2017) and Ip and Cutler (2020) have shown that Mandarin Chinese listeners are equally good at using intonation to predict an upcoming focus as native English listeners, which suggests that tone and intonation can be integrated in online tasks. More research is needed to determine the conditions that make intonational processing harder for Mandarin speakers and those that are not problematic. Our data show that pitch accent contrasts that sound distinct to Mandarin Chinese listeners can be easily imitated/acquired in the L2.

From a broader perspective, our data show that CLI is the decisive factor in the acquisition of pitch accent contrasts. For both learner groups (i.e., for learners whose language background is either a tone language or another intonation language), specifics of the native language are able to explain the realization of the contrasts in the L2. The influence of the tonal background (Mandarin) was already discussed in the preceding paragraph. We will focus on the Italian system to understand the nature of transfer better. The Italian intonational inventory consists of two monotonal (L* and H*) and seven bitonal accents: H + L*, H* + L, L + H*, L + ¡H*, L+ < H*, L* + H, L*+ > H (Gili Fivela et al., 2015). Note that these accent types occur in a number of varieties across Italy, including varieties of northern and central Italy where our speakers came from. We briefly describe these pitch accents to explain the nature of CLI that can be expected. In L + H*, the H is aligned in the middle or at the end of the stressed syllable. In L + ¡H*, the high tonal target is also aligned at the end of the stressed syllable, but in addition is described as superhigh. In L+ < H*, the starred tone is aligned in the post-stressed syllable or even later. For these three “L + H*-variants,” the alignment of the L tone is not described and may therefore not be considered relevant for the characterization of an accent. As evident from schematic representations in Gili Fivela et al. (2015, p: 148), the L alignment seems to be at the beginning of the stressed syllable. In L* + H and L*+ > H (i.e., the “L* + H-variants”), there is a fall to the stressed syllable before the accentual rise. Other than that, both tonal targets are aligned in the stressed syllable. In terms of a potential mapping from L1 to L2 categories, Italian L + H* could be mapped onto German L + H*, Italian L + ¡H* on German (LH)* – if we assume that a superhigh peak results in a steeper slope – and Italian L+ < H* on German L* + H. Given that such a mapping is possible, Italian learners ought to be well equipped to imitate the German accentual contrast. However, we observe some differences in accent realization: In the non-merger contrast [(LH)* vs. L* + H], Italian speakers mainly differed in the post-stressed syllable from the German natives, maybe owing to the fact that there are no rising accents with a late peak [the only rising accents (L* + H and L*+ > H) are preceded by a fall]. The merger contrast [(LH)* vs. L + H*], in turn, was completely mapped onto one contour in Italian learners, with no difference between contours. This finding cannot readily be explained by the Italian phonological system. If anything, it is possible that the actual phonetic alignment differs between Italian and German and that Italian learners of German were not able to perceive a difference between the two accents. We will have to leave this open question to be tested in future research. What is even more important, however, is the comparison of the two learner groups. Here, Italian learners did not differ from Mandarin Chinese learners in the merger contrast, but were closer to the native German speakers in the non-merger contrast.

Contrary to what was predicted by H1 and H2, our data do not suggest that speakers of a tone language may acquire intonational contrasts *generally* more easily or with greater difficulty than speakers of an intonation language. The deviations from the target group realized by learners could – by and large – be explained by the properties of their native language, i.e., CLI (H3). In other words, what we observe is transfer from the L1 to the L2 – a phenomenon that has been shown to occur in various different L2 studies for both segmental (e.g., Flege et al., 1997; Abrahamsson and Hyltenstam, 2009; Hattori and Iverson, 2009; Schmid et al., 2014) and suprasegmental aspects (e.g., Mennen, 1998; Atterer and Ladd, 2004; Arvaniti et al., 2006; Zahner and Yu, 2019; Manzoni-Luxenburger, 2021). As already pointed out by Flege and Bohn (2022), it is difficult to operationalize the perceived phonetic (dis)similarity between L1 and L2 categories on the segmental level. This may hold true even more so for the comparison of tonal and intonational contrasts on the supra-segmental level. We used judgements by L1 Chinese informants without knowledge of German on the distinction between contrasts, resulting in a merger (similar) and non-merger (dis-similar) contrast. Yet, our informants had difficulties mapping the accentual realizations in an unknown L2 onto lexical tone sequences. One possibility to overcome this issue and arrive at a measure of (dis)similarity between L1 and L2 categories would be to have listeners judge how close L2 realizations of pitch accents of the noun (e.g., *Mandalas*) are to trisyllabic tone sequences. However, this kind of data would also rely on metalinguistic judgments. We believe that the imitation paradigm is better-suited to determine phonetic (dis)similarity, as it provides a more direct window into the representations of developing accent categories in the L2. Nevertheless, it stands to reason whether the categories of a tone language might be *per se* more distant than the pitch accents of any other intonation language.

A further factor that may explain differences between Mandarin Chinese and Italian learners (but not the differences in the realization of the two kinds of contrasts for Mandarin Chinese learners) is lexical proximity – the two low-proficient learner groups were matched in proficiency (both when measured in DIALANG and in perceived foreign accentedness). The lexical items, which were chosen to contain mostly sonorant segments, may have been more familiar to Italian than to Mandarin Chinese participants. In particular, the drink “Malibu,” the soccer team position “Libero” and the coloring picture “Mandala” are German-Italian cognates and hence exist in the Italian lexicon as well, while they do not exist in Mandarin Chinese. Due to their comparably low lexical frequency^13^, it is very unlikely that they are part of the (average) L2 lexicon, so that they must be considered novel words for Mandarin Chinese learners. However, it is not entirely clear how the presence of cognates could have affected the imitation task: On the one hand, the presence of cognates may allow Italian participants to focus on prosody more. For instance, Italian speakers are well able to imitate an alignment pattern of a different Italian variety (D’Imperio et al., 2014; but note that the task may have been easier than the task in the present study because participants did not have to wait before initiating the imitation). On the other hand, the presence of cognates may strengthen L1 transfer, as has been shown for the production of VOT in Spanish learners of English (Amengual, 2012) or phonological /s/ in Spanish-English bilinguals (Brown and Harper, 2009). Note, however, that the main argument of this paper concerns the realization of the two kinds of contrasts for Mandarin Chinese learners, which is unaffected by these lexical considerations, as the items are assumed to be equally unknown to both Mandarin Chinese groups.

The pitch accent contrast between (LH)* and L* + H (non-merger contrast) was acoustically more pronounced than the contrast between (LH)* and L + H* (merger contrast) in all groups. Actually, the terms “non-merger” and “merger” contrast were chosen based on the way Mandarin Chinese speakers perceive the pitch accents. It seems, however, that the merger contrast, for which the pitch peak was aligned with the stressed syllable for both accents, was subtle in terms of f0 differences overall (even for native speakers, Zahner-Ritter et al., 2022). Contexts in which the (LH)* accent occurs in German are attitudinally loaded utterances (rhetorical questions, *cf.* Braun et al., 2019), utterances that signal surprise (Kohler, 2005; Wochner, Forthcoming, for exclamatives) or utterances that mainly signal surprise, aversion, or correction (for declaratives, see Zahner-Ritter et al., 2022). In rhetorical questions and exclamatives, the (LH)* accent is accompanied by further prosodic modification, in particular lengthening and non-modal voice quality (Braun et al., 2019; Wochner, Forthcoming), not necessarily co-occurring with the accented word but occurring across the utterance. Listeners, in turn, do not only use information on the pitch accent type when identifying rhetorical questions, but additionally use durational and voice quality cues (Kharaman et al., 2019). Intensity and voice quality could not be analyzed with the present data set because of remote data collection; the fact that participants used their own microphones led to great differences in recording quality, which does not lend itself to further phonetic analysis.

It may hence be the case that we are currently overlooking critical aspects when focusing on the analysis of f0 contours only. In future studies, we plan to investigate how tone in the L1 affects the production of pitch accents in their entirety, including durational aspects, voice quality, and intensity in the entire utterance. Post-hoc durational analyses of the object noun of the present data show no effects of group or intonation condition on the duration of the first, stressed syllable and on the last syllable. For the second syllable, however, there was a significant interaction between language group and condition: German and Italian participants did not modulate duration as a function of intonation condition. Mandarin participants (in particular low-proficient speakers), on the other hand, produced longer syllable durations in the L* + H condition than in the other two intonation conditions. This lends further evidence to the observation that some low-proficient Mandarin learners of German may produce a different metrical structure compared to Italian learners or German native speakers.

The present study focused on the phonetic and phonological acquisition of pitch accent contrasts. A further desideratum is to test whether learners can actually use the contrasts in appropriate contexts, which is a key requisite for correct acquisition and successful communication (*cf.* Mennen, 2015).

### Proficiency

In this paper, we compared low- and high-proficient Mandarin Chinese learners of German. For the non-merger contrast, the realization of the contrast in the high-proficient group was closer to native speakers than in the low-proficient group; for the merger-contrast, no effect of proficiency was observed.^14^ The beneficial effect of proficiency for the non-merger contrast is in line with previous studies (e.g., Baker, 2010; He et al., 2012; Graham and Post, 2018; Shang and Elvira-García, 2022) and experience has been considered in models of L2 acquisition (Flege, 1995; Best and Tyler, 2007; Mennen, 2015; Flege and Bohn, 2022), see Piske (2007) and Tyler (2019) on the relevance of experience in a classroom setting. Importantly, proficiency effects were not observed across the board in our data. In the merger contrast [(LH)* vs. L + H*, which was perceived as similar by Mandarin Chinese listeners], both low- and high-proficient Mandarin learners deviated equally from the native speakers’ productions. It hence seems that CLI was stronger than proficiency and may have overwritten the beneficial effect of proficiency. Here, it might be interesting to test an immersed learner group to see whether in such a group, the native Mandarin pattern may be inhibited by German to arrive at target-like productions. It is also conceivable, however, that the realization of the merger contrast, (LH)* vs. L + H*, was already very target-like in the low-proficient group, leaving no room for a positive effect of proficiency (ceiling effect). A way to mathematically model the effect of proficiency is to use the PENTA model (Xu, 2005) and to either remove the targets for tones or to reduce the strength of target approximation with increasing proficiency.

The participants were grouped into high- vs. low-proficient speakers according to a lexical task (DIALANG), which has been argued to be suited to assess L2 proficiency (Alderson, 2005). Furthermore, it has been shown to correlate well with self-rated proficiency and general proficiency factors such as age of onset, language use, and language preference (Lloyd-Smith et al., 2020). To tap more deeply into phonetic/phonological aspects for the current set of sentences, we further solicited perceived foreign accent ratings (Levi et al., 2007; Hopp and Schmid, 2013). Interestingly, for the current data set, the DIALANG scores correlated only weakly with perceived foreign accent ratings, *r* = −0.36 (*t* = −2.29, *df* = 34, *p* = 0.03). It is hence possible that general language skills (such as vocabulary development) are partly dissociated from phonetic and phonological processing. For our purposes, we used the DIALANG score as a measure of proficiency, since otherwise, the argument would have become circular (we cannot exclude that foreign accent ratings are influenced by the intonational realization of the utterances – the very aspect we intend to study). In future research it may be promising to include proficiency as a continuous rather than a categorical variable in the statistical modeling (*cf.* Porretta et al., 2016) to derive a more fine-grained picture. We also leave the factors beyond proficiency, such as motivation, personality, attitude toward the L1 and L2, as well as language aptitude for future research, *cf.* Jilka (2009).

## Conclusion

Low- and high-proficient Mandarin Chinese learners of German imitated a three-way pitch accent contrast in an intonational L2. The decisive factor in the realization of pitch accent contrasts was whether the pitch accents were perceived as dissimilar [non-merger contrast, here (LH)* vs. L* + H] or similar [merger contrast, here (LH)* vs. L + H*]. Higher proficiency led to more target-like productions, at least in the non-merger contrast. Comparisons with imitations of Italian learners of German showed that native language experience with a tone language neither yields a general disadvantage in the acquisition of L2 pitch accent contrasts nor a general advantage, but clearly exhibits crosstalk between lexical tone and intonation (which can be best interpreted as CLI).

## Data availability statement

The datasets presented in this study can be found in online repositories. The names of the repository/repositories and accession number(s) can be found at: http://doi.org/10.17632/w293n86sjr.2.

## Ethics statement

The studies involving human participants were reviewed and approved by IRB Konstanz, 05/2021. The patients/participants provided their written informed consent to participate in this study.

## Author contributions

KZ-R, TZ, ME, and BB contributed to the conception of Experiment 1. ME and TZ contributed to the conception of Experiment 2 and carried out the experiment and data annotation for Experiments 1 and 2, respectively. KZ-R and BB led the statistical analyses. All authors contributed to the article and approved the submitted version.

## Funding

The research was funded by the DFG as part of research unit “Questions at the Interfaces” (FOR 2111, P6, PIs Bettina Braun and Nicole Dehé), grant numbers BR 3428/4–1,2 and DE 876/3–1,2. We also acknowledge funding from the Trier Center for Language and Communication (TCLC, Patterns, *Forschungsinitiative Rheinland-Pfalz 2019–2023*).

## Conflict of interest

The authors declare that the research was conducted in the absence of any commercial or financial relationships that could be construed as a potential conflict of interest.

## Footnotes

^1^Cross-linguistic comparisons mostly exist with regard to the acquisition of lexical tone languages (e.g., Gandour, 1983; Chiao et al., 2011; Qin and Mok, 2011; Xu and Mok, 2012, 2014; Braun et al., 2014); for an overview and methodological considerations for native and non-native tone perception see Best (2019).

^2^The typological status of languages such as Japanese or Swedish in which lexical pitch accents occur on certain words but not on others (pitch-accent languages) is not of concern for the present paper. Likewise, languages that use intonation to (mostly) mark syntactic phrasing (e.g., French, Japanese, Korean, Bengali, Urdu) are not dealt with here (*cf.* Jun, 2005, for overview).

^3^The neutral tone is typically realised with falling pitch when occurring after Tone 1, 2, and 4, while it is realised with rising pitch after the low-dipping Tone 3 (Yip, 2002, see also Zhang et al., 2022). From a theoretical perspective, the status of lexical tone has been controversially discussed in the linguistic literature and it has not entirely been resolved whether it serves segmental or suprasegmental functions (Yip, 2002; Hyman, 2011; Best, 2019).

^4^Other options to mark information status, information-structure, illocution type, and discourse organisation are word order or particles. In addition, studies have shown that in several tone languages, pitch accents only play a minor role beyond the lexical tone level (Laniran and Clements, 2003; Connell, 2017) and that crosstalk between lexical tone and intonation in tone language may be rather limited (Zerbian, 2016). However, there is also evidence from some other tone languages that intonational effects may be phonetically layered on existing lexical tones, hence revealing crosstalk between the two levels (Xu, 1999; DiCanio et al., 2018).

^5^Note that Mandarin and Cantonese listeners also show a great sensitivity to segmental cues, which may even outweigh tonal cues (e.g., Taft and Chen, 1992; Cutler and Chen, 1997).

^6^Mennen argues that it is crucial to determine whether instances of the L2 category are interpreted as members of the L1 category (Mennen, 2015). Ideally, we wanted to know whether the pitch accents could be transcribed as a certain tone sequence, but this task was too meta-linguistic and caused confusion for native Mandarin speakers (without explicit linguistic knowledge). We instead used the judgements of our informants on the perceived (dis)similarity between the contrasts as a measure to set up the merger vs. non-merger contrast.

^7^The DIALANG test served as a proxy for assessing the development on all linguistic areas. The DIALANG test scores have been found to correlate well with self-rated proficiency and general proficiency factors such as age of onset, language use, and language preference (Lloyd-Smith et al., 2020).

^8^In the overall data set (*N* = 55), there were more high-proficient participants from the North and vice versa for the South (Zhao, 2022).

^9^The source recording did not have an effect in the German data (Zahner-Ritter et al., 2022) and is hence not considered here either.

^10^Note that in both contrasts, the two Mandarin Chinese groups started with higher f0 than the German group (see Panels A in Figures 5, 6). This difference in pitch scaling is not very relevant, however. In our analysis, we did not compare accent realizations across groups, but the realization of accentual *contrasts* across groups. This allows us to abstract from the generally higher pitch level in the learners’ productions prior to the accent.

^11^Unlike German, it lacks post-focus deaccentuation (Swerts et al., 2002, for experimental evidence), but this difference is not relevant as we are dealing with utterances that have the nuclear accent on the last word.

^12^The confidence intervals are broader for the Italian group, probably owing to the smaller number of participants.

^13^The words are not even listed in the CELEX corpus (Baayen et al., 1993); dlexDB (Heister et al., 2011) reveals a very low frequency, ranging from 1 for "Mandalas" (0.43 occurrences per million) to 116 for "Melanie" (50.43 o.p.m.).

^14^*Post-hoc* analyses suggested by one of the reviewers indicated that the better performance of the high-proficient group was carried mainly by the speakers from the northern parts of China. Whether or not this is an effect of contact language or due to other aspects (e.g., proficiency in another intonation language, such as English) needs to be left for future research that explicitly manipulates *region* as a factor. In our case, speakers from different regions were chosen to better generalize the results, but regional differences were not our interest.
