# Supplementary material for: How experience with tone in the native language affects the L2 acquisition of pitch accents
Source: Front Psychol. 2022 Aug 19;13:903879. doi: 10.3389/fpsyg.2022.903879 (PMC9437707; doi:10.3389/fpsyg.2022.903879)
Supplement: Supplementary file 1 [file Data_Sheet_1.pdf]

Supplementary Material

1 Supplementary Material 1: Experiment 1: Overview table of the Mandarin participants

Supplementary Material S.1.1 provides a comprehensive overview of background information of the participants. The basic information and linguistic experience (with German and English) are presented across two proficiency groups and two region groups. DIALANG scores range from 0 (no knowledge of German) to 75 (excellent knowledge of German). Foreign accent ratings range from 1 (no foreign accent) and 6 (strong foreign accent). Time of learning German and living in Germany is presented in years, and the last two columns in numbers of the participants. **Note:** Dataset and analysis scripts can be found here: http://dx.doi.org/10.17632/yhv7nmjmgf.2.

**Proficiency**	**Region**	**Age (mean and (sd))**	**DIALANG score (mean and (sd))**	**Foreign accent rating (mean and (sd))**	**Time of German learning (mean and (sd))**	**Time of residence in Germany (mean and (sd))**	**Exposure to English before age 4**	**Self-rated English proficiency**
Low-proficiency group	north	23.3 (3.4)	45.6 (7.2)	4.8 (1.8)	1.7 (1.5)	0.5 (0.85)	2	1 x A1/A2, 3 x B1/B2, 3 x C1/C2
	south	21.6 (2.2)	45.1 (5.9)	4.9 (1.3)	1.6 (0.8)	0.79 (1.2)	2	2 x B1/B2, 5 x C1/C2
High-proficiency group	north	23.6 (4.0)	60.7 (3.9)	3.8 (1.5)	6.1 (2.9)	2.2 (2.4)	3	3 x B1/B2, 4 x C1/C2
	south	23.9 (1.1)	59.1 (3.2)	4.2 (1.4)	4.5 (1.7)	1.6 (1.9)	1	6 x B1/B2, 1 x C1/C2
